# Differential IL-13 Production by Small Intestinal Leukocytes in Active Coeliac Disease versus Refractory Coeliac Disease

**DOI:** 10.1155/2013/939047

**Published:** 2013-04-15

**Authors:** Sascha Gross, Roy L. van Wanrooij, Petula Nijeboer, Kyra A. Gelderman, Saskia A. G. M. Cillessen, Gerrit A. Meijer, Chris J. J. Mulder, Gerd Bouma, B. Mary E. von Blomberg, Hetty J. Bontkes

**Affiliations:** ^1^Department of Pathology, VU University Medical Center, P.O. Box 7057, 1007 MB Amsterdam, The Netherlands; ^2^Department of Gastroenterology, VU University Medical Center, P.O. Box 7057, 1007 MB Amsterdam, The Netherlands

## Abstract

A small fraction of coeliac disease (CD) patients have persistent villous atrophy despite strict adherence to a gluten-free diet. Some of these refractory CD (RCD) patients develop a clonal expansion of lymphocytes with an aberrant phenotype, referred to as RCD type II (RCDII). Pathogenesis of active CD (ACD) has been shown to be related to gluten-specific immunity whereas the disease is no longer gluten driven in RCD. We therefore hypothesized that the immune response is differentially regulated by cytokines in ACD versus RCDII and investigated mucosal cytokine release after polyclonal stimulation of isolated mucosal lymphocytes. Secretion of the T_H_2 cytokine IL-13 was significantly higher in lamina propria leukocytes (LPLs) isolated from RCDII patients as compared to LPL from ACD patients (*P* = 0.05). In patients successfully treated with a gluten-free diet LPL-derived IL-13 production was also higher as compared to ACD patients (*P* = 0.02). IL-13 secretion correlated with other T_H_2 as well as T_H_1 cytokines but not with IL-10 secretion. Overall, the cytokine production pattern of LPL in RCDII showed more similarities with LPL isolated from GFD patients than from ACD patients. Our data suggest that different immunological processes are involved in RCDII and ACD with a potential role for IL-13.

## 1. Introduction

Coeliac disease (CD) is an autoimmune enteropathy that is triggered by the gliadin fraction of dietary gluten peptides [1]. The immune processes in CD have been widely studied and it is commonly accepted that in CD innate and adaptive immune responses are part of the pathogenesis [2]. Gliadins can exert direct toxic effects by binding to epithelial cells, resulting in the production of IL-15 and TNF*α* [3–5]. IL-15 upregulates natural-killer receptors on intra-epithelial cytotoxic T lymphocytes as well as their ligands on epithelial cells, which leads to enhanced apoptotic killing of epithelial cells [6]. The main pathogenic mechanism of CD, however, is believed to be a gluten-specific T_H_1-mediated response resulting in an overexpression of IFN*γ* in the (intra) epithelial compartment [7]. IFN*γ*, together with TNF*α*, enhances the expression of transglutaminase-2 (TG2) [8]. TG2 binds and deamidates gliadin peptides, which leads to a better presentation of gliadin peptides to specific T_H_ cells and a subsequent stronger gliadin-specific immune response with even higher amounts of IFN*γ* [9, 10]. Although the exact mechanism is unknown, evidence exists that the overexpressed IFN*γ* ultimately leads to the mucosal damage found in CD [11, 12]. More recently, the proinflammatory cytokine IL-17A has been found to play an important role in coeliac pathology as well [13]. Despite a predominant proinflammatory cytokine profile in active CD, also expression of the regulatory cytokine IL-10 is found, possibly limiting the production of proinflammatory cytokines [14]. Indeed, in a pilot phase I study, treatment with recombinant IL-10 did induce some relief of symptoms in a minority of patients but IL-10 treatment did not lead to mucosal recovery [15].

In contrast to uncomplicated CD, less is known about the pathology of refractory coeliac disease (RCD) [16]. RCD is a complication of CD in which patients despite following a strict gluten-free diet (GFD) do not recover from symptoms and mucosal lesions. RCD type II (RCDII) is characterized by a significant (>20%) aberrant intraepithelial T lymphocyte (IEL) population in the small intestinal mucosa. These aberrant IEL lack T-cell-specific surface markers, that is, T-cell receptor (TCR), CD3, CD4, and CD8, but express cytoplasmic CD3. Clonal expansion of these aberrant IEL is thought to be responsible for the occurrence of enteropathy-associated T-cell lymphoma (EATL), which occurs in 60%–80% of RCDII patients within 5 years [17]. Similarly to uncomplicated, active CD, IL-15 and IFN*γ* are reported to be enhanced in RCD; however it is unclear whether they play significant roles in the pathogenesis of RCD [18, 19]. TNF*α* may play a role in RCD, since some RCD cases have been described where anti-TNF*α* therapy has shown to have a beneficial effect [20, 21]. IL-17A, IL-13, and IL-5 have not yet been investigated in RCD.

As in RCD the immunological trigger gliadin is absent, we hypothesized that the cytokine profile of IEL and lamina propria leukocytes (LPL) is altered as compared to the gliadin-driven immune response in ACD. Therefore, we measured protein levels of the proinflammatory cytokines TNF*α*, IFN*γ*, and IL-17A, the T_H_2 cytokines IL-13 and IL-5, and the regulatory cytokine IL-10, in supernatants of polyclonally stimulated leucocytes from biopsies of uncomplicated CD and RCD patients.

## 2. Patients and Methods

### 2.1. Patients

Consecutive patients (*n* = 20) were included in our study that visited our outpatient clinic for CD or RCD follow-up. Biopsies were taken for diagnostic purposes and cells remaining from the diagnostic procedure were used for our experiments. The study protocol adhered to the guidelines set by our institutional ethical committee. Patients with concomitant complications such as ulcerative jejunitis or autoimmune enteropathy and patients with collagenous sprue were excluded. Active CD (ACD) was diagnosed according to current guidelines for adult CD [22], that is, if biopsies showed increased numbers of intraepithelial lymphocytes, crypt hyperplasia, and villous atrophy together with antibodies against transglutaminase-2 (TG2A) and endomysium. CD patients were prescribed a gluten-free diet (GFD) and were considered recovered when TG2A levels normalized and when follow-up biopsies showed no villous atrophy anymore (Marsh 0–II; GFD patient group). Adherence to a GFD was confirmed by a dietitian and absence of TG2A in serum. Follow-up biopsies were taken in order to confirm histological recovery or when CD symptoms persisted and RCD was suspected.

Patients were diagnosed with RCD when malabsorption symptoms and histological abnormalities persisted or recurred despite strict dietary adherence (as confirmed by the disappearance of TG2A and EMA) and after exclusion of other intestinal diseases. RCDII was diagnosed, if an aberrant IEL population (CD3^−^, intracellular CD3^+^, CD7^+^) occurred with a frequency of more than 20% of all IEL [23]. Since the distinction between RCDI and slow responders on a GFD can only be done after a long-term follow-up, patients with suspected RCDI were excluded and only patients with RCDII were included in this study. RCDII patients were treated with autologous stem cell transplantation (SCT), 6-thiogunidine (6-TG), cladribine, or entocort; one patient was analysed prior to treatment (Table 2). Similarly to CD patients, RCDII patients were considered recovered, when villous atrophy was absent after therapy. 

### 2.2. Cell Cultures and Cytokine Measurement

Small intestinal biopsies were separated into epithelial layer and lamina propria by incubation in PBS containing DDT and EDTA in a 37°C shaking water bath for one hour as previously described [24]. IEL were washed and collected in ice-cold PBS-BSA 0.1%. The remaining lamina propria was incubated for 2 h in PBS with 10% FCS and 0.16 U/mL collagenase (Collagenase A, Roche). After incubation the biopsies were passed through a sterile 100 *μ*m and filtered through a sterile 40 *μ*m mesh. Cells were then washed and collected in ice-cold PBS containing 0.1% BSA. IEL and LPL were incubated for at least 15 min. with magnetic beads linked to anti-CD45 antibodies (MACS human-CD45 MicroBeads, Miltenyi Biotec). CD45-positive cells (leukocytes) were separated on a magnetic column (MACS MS column, Miltenyi Biotec), collected, and divided over two (IEL) or three (LPL) wells of a 96-well cell-culture plate: IEL: (1) unstimulated, (2) stimulated with 50 ng/mL PMA, 1 *μ*g/mL ionomycin, and 50 ng/mL LPS; LPL: (1) unstimulated, (2) stimulated with 50 ng/mL PMA and 1 *μ*g/mL ionomycin, and (3) stimulated with 50 ng/mL LPS. Each well contained the cells of approximately 2 biopsies in a total volume of 100 *μ*L. After 24 hour incubation at 37°C and 5% CO_2_, supernatants were collected, frozen, and stored at −20°C until analysed. Cytokine levels of TNF*α*, IL-17A, IL-13, IL-10, and IL-5 were determined using a multiplex bead assay (Cytometric Bead Assay, BD). IFN*γ* was measured using a commercially available ELISA kit (PeliKine compact human IFN*γ*, Sanguin).

### 2.3. FACS Analyses

Cell subsets, that is, CD4^+^ and CD8^+^ T cells, CD3-CD16/56^+^ NK cells, and CD19^+^ B-cells, were determined by multicolour FACS analysis using CD3-FITC, CD8-PE, CD45-PerCP, and CD4-APC and CD3-FITC, CD16/56-PE CD45-PerCP, and CD19-APC antibody conjugates, respectively (Multitest, BD). Aberrant IEL were analysed by surface CD3, CD52, and CD45 followed by cytoplasmic staining of CD3 after cell permeabilization (Cytofix/CytoPerm Plus kit, BD Biosciences). All analyses were performed on lymphocytes, based on bright CD45 staining and low side scatter (SSC). Aberrant T cells were defined as CD52^+^ cytoplasmic CD3^++^ and surface CD3 negative cells. Total numbers of IEL (cell harvest) were determined using FACS tubes containing a fixed number of reference beads (Trucount tubes, BD).

### 2.4. Statistical Analyses

Differences in cytokine levels were tested with the Mann-Witney *U* test. Difference in sex distribution was tested with the chi-square test. Differences in age, cell count, and cell type ratios were tested with the student's *t*-test. Correlation coefficients were calculated with a two-sided Pearson's correlation.

## 3. Results

### 3.1. Patient Characteristics and Composition of Leukocyte Infiltrates

A total of 20 patients were included in our study: 4 patients with active coeliac disease (ACD), 7 on a gluten-free diet (GFD), and 9 patients with RCDII. RCDII patients tended to be older at the time of cytokine analysis than ACD patients (*P* = 0.07, Table 1). The follow-up time of GFD patients was at least 8 months, and that of RCDII patients at least 2 years since the start of the gluten-free diet (data not shown).

Five of the RCDII patients had villous atrophy. One of these patients was not treated and four retained villous atrophy despite treatment (Table 2). Of the four patients that recovered histologically after treatment, one was treated with SCT, and the other three with cladribine.

Cell yield (total number of isolated IEL) did not differ significantly between groups. The median cell yield was highest in ACD patients with 28,500 cells per biopsy compared to GFD (16,000 cells per biopsy) and RCDII (19,100 cells per biopsy). Due to large variation, however, no significant difference in cell yield was observed between groups. The percentage of CD3-positive IEL, mostly CD8^+^ T-cells, was significantly lower in RCDII patients compared to GFD and ACD, which is due to the high percentage of aberrant IEL found in RCDII patients (Table 1). NK cell frequencies in IEL were generally low (Table 1) and B-cells were absent (data not shown). In the LPL fraction NK cell and B-cell frequencies were below 10% in all groups (Table 1).

### 3.2. Cytokine Levels in IEL

**Figure 1:**
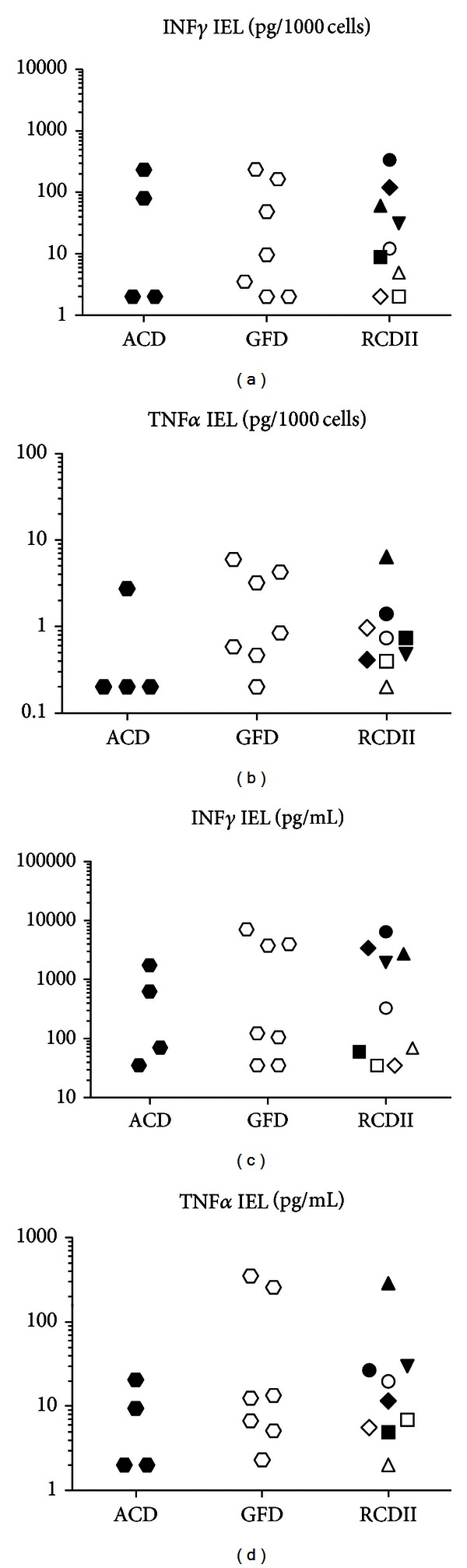
Production of INF*γ* and TNF*α* by IEL from active CD patients (ACD), patients on a gluten-free diet (GFD), and refractory CD type II (RCDII) patients after PMA/ionomycin/LPS stimulation. RCDII patients with villous atrophy (closed symbols); RCDII patients without villous atrophy (open symbols); for individual characteristics see Table 2. ((a), (c)) IFN*γ* and ((b), (d)) TNF*α* production. ((a), (b)) Production per 1000 IEL or ((c), (d)) per mL per two biopsies.

Stimulation of IEL overall resulted in low cytokine levels, probably due to the generally low numbers of leukocytes present in the epithelial layer. Only IFN*γ* and TNF*α*, both known to be increased in the duodenum of CD patients, reached detectable levels in IEL. In order to analyse whether IEL numbers may influence possible differences in cytokine levels between the groups, the amount of cytokine was divided by the number of IEL that were isolated from biopsies. No significant differences could be found between ACD and RCDII patients whether the amount of cytokine per 1000 IEL (Figures 1(a) and 1(b)) or the amount of cytokine per two biopsies (Figures 1(c) and 1(d)) was analysed. IFN*γ* production was not lower in GFD patients as compared to ACD patients. However, in the RCDII group, IEL-derived IFN*γ* production was the highest in patients with persisting villous atrophy (Figures 1(a) and 1(b), closed symbols).

### 3.3. Cytokine Levels in LPL

LPLs were stimulated with either PMA/ionomycin to trigger all the leukocytes or LPS to trigger antigen-presenting cells (APC) only. After LPS stimulation most cytokines were undetectable and only low levels of IFN*γ* and TNF*α* were detectable in a minority of the patients. For both IFN*γ* and TNF*α* no differences could be observed between groups after LPS stimulation (data not shown). As in general the levels were 50- to 500- fold lower after LPS stimulation as compared to PMA/ionomycin stimulation, IFN*γ* and TNF*α* production after PMA/ionomycin will be mostly lymphocyte rather than APC derived.

**Figure 2:**
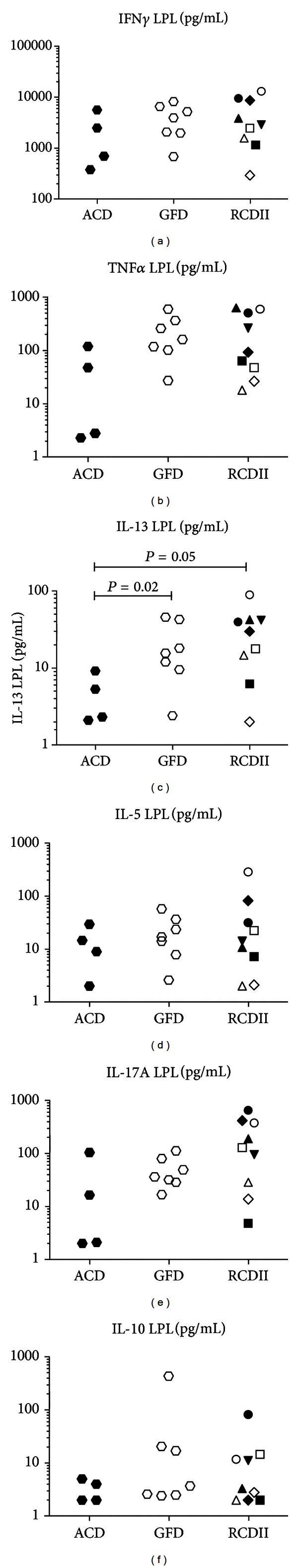
Production of (a) IFN*γ*, (b) TNF*α*, (c) IL-13, (d) IL-5, (e) IL-17, and (f) IL-10 by LPL from active CD patients (ACD), patients on a gluten-free diet (GFD), and refractory CD type II (RCDII) patients after PMA/ionomycin stimulation. RCDII patients with villous atrophy (closed symbols); RCDII patients without villous atrophy (open symbols). Groups were compared using the Mann-Whitney *U* test. *P* values are shown for significant differences.

**Figure 3:**
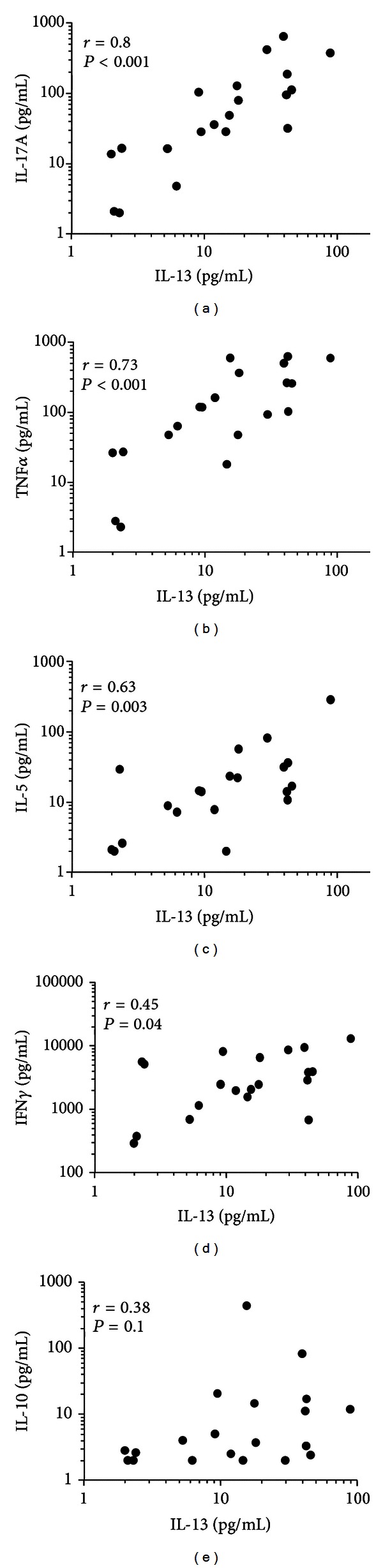
Correlation between (a) IL-13 and IL-17A, (b) TNF*α*, (c) IL-5, (d) IFN*γ* and (e) IL-10 production in all groups. Correlations were tested with a two-sided Pearson correlation.

In contrast to LPS, stimulation of LPL with PMA/ionomycin resulted in detectable cytokine levels. RCDII patients who were treated within 6 weeks before the biopsy was taken appeared not to be different in terms of cytokine production from patients who were treated more than 6 weeks before the biopsy was taken (Figure 2 and Table 2). However, levels of most cytokines (IFN*γ*, TNF*α*, IL-13, and IL-17A) tended to be the highest in patients with persisting villous atrophy (Figure 2, closed symbols). Similar to the IEL results, IFN*γ* production by LPL was comparable between ACD and RCDII patients and IFN*γ* production was not reduced in GFD patients compared to ACD (Figure 2(a)). IL-13 responses were higher in RCDII when compared to ACD patients but were also higher in GFD as compared to ACD (Figure 2(c)). Since IL-13 production was significantly increased in RCDII patients as compared to ACD patients, we analysed the coexpression of IL-13 and the other cytokines by calculating correlation coefficients for all IL-13 cytokine pairs. IL-13 release correlated the strongest with IL-17A and TNF (*r* = 0.80 and *r* = 0.73, resp.; both *P* < 0.001; Figures 3(a) and 3(b)). Weaker correlations were observed with IL-5 and IFN*γ* (*r* = 0.63, *P* = 0.003 and *r* = 0.45, *P* = 0.04, resp.; Figures 3(c) and 3(d)), while there was no significant correlation between IL-13 and IL-10 (*r* = 0.38, *P* = 0.10; Figure 3(e)).

## 4. Discussion

In this study we tested the hypothesis that the local cytokine profile would be different in gluten-driven ACD as compared to gluten-independent RCDII. This was investigated by analysing the capacity of LPL and IEL isolated from the duodenum of ACD and RCDII patients as well as from patients successfully treated with a gluten-free diet to produce IFN*γ*, TNF*α*, IL-17A, IL-13, IL-5, and IL-10. IL-15 was not analysed as it is not well secreted and unstable [25].

IFN*γ* production has been extensively studied in ACD and GFD. While IFN*γ* has been considered to play an important role in enterocyte destruction in ACD, several studies have shown that IFN*γ* levels are not reduced in GFD [26, 27]. This is in line with our findings that show no difference in the capacity to produce IFN*γ* between IEL/LPL from ACD and GFD patients. Here, we also show that there is no increase in IFN*γ* production in RCDII patients. This suggests that the capacity of IEL/LPL to produce IFN*γ* appears not to be solely dependent on an ongoing gluten-driven immune response.

In contrast to our findings here, levels of TNF*α* protein have been found to be elevated in lamina propria and epithelium of ACD patients and decreased after a GFD [28, 29]. However, there are important methodological differences between the present and these previous studies. While we used PMA and ionomycin stimulation to analyse the capacity of the IEL/LPL to produce particular cytokines, the above mentioned studies used RT-PCR analysis or immunohistochemistry to analyse cytokine mRNA levels or protein without prior stimulation. This suggests that the capacity of IEL and LPL to produce TNF*α* may be similar in ACD and GFD while the current production at the time of biopsy may be reduced in GFD. 

Although there is a considerable overlap between the groups, the capacity of LPL to produce IL-13 and IL-17A seems to be lower in ACD as compared to RCDII and GFD, which reached statistical significance for IL-13 when analysed individually. In paediatric ACD patients, lower numbers of mucosal T cells with the capacity to produce IL-17A were observed as compared to controls. It was suggested that the relative lack of IL-17A producing T cells may affect the homeostasis of the epithelial layer and contribute to increased intestinal permeability [30]. In our dataset this was less apparent; however, in a subset of RCDII patients (particularly those with persistent villous atrophy despite treatment) high levels of IL-17A were detected after polyclonal stimulation and in only one of the ACD patients, suggesting a differentially driven IL-17A response in treatment-resistant RCDII patients. This increased capacity of LPL to produce IL-17A in treatment-resistant RCDII may be related to the continued inflammation and risk of EATL development, as IL-17A is involved in chronic inflammation as well as in tumour formation [31]. 

To the best of our knowledge this is the first study that investigated local IL-13 levels in CD and RCD. In our experiments we found higher IL-13 production in RCDII patients as compared to ACD patients. IL-13 production capacity was also higher in GFD patients compared to ACD. Although IL-13 is mainly associated with airway pathology, it also has an important role in gut defence and inflammation [32]. In ulcerative colitis the high levels of IL-13 are shown to be derived from variant CD1d-restricted NKT cells and IL-13 has been shown to have a toxic effect on colonic epithelial cells [33, 34]. IL-13 has also been shown to be produced by NK cells as part of an innate response [35]. This is in line with the high levels of IL-13 found in RCDII where antigenic stimulation by gluten is lacking. The higher IL-13 production was not related to NK cell frequencies; whether the IL-13 produced is NK or variant NKT cell derived remains to be investigated. IL-13 production capacity was not only correlated to IL-17A production but also to the other T_H_1 and T_H_2 cytokines, but not to the regulatory cytokine IL-10, which is in line with a proinflammatory role for this cytokine.

IL-13 has been shown to have direct cytotoxic effects on epithelial cells. It is, therefore, intriguing to speculate why there is an increased capacity of LPL to produce IL-13 in both patients on a successful GFD and RCDII patients. Differential expression of the receptors on epithelial cells as has been described for the IL-15 receptor [36] as well as regulatory cytokines not measured here (TGF*β*) or contact-dependent regulation by regulatory cells may play a role. Although the difference between RCDII and ACD was only statistically significant for the IL-13 production capacity, the production pattern of the other cytokines was comparable, and the overall cytokine profile of LPL in RCDII showed more similarities with LPL from GFD patients than from ACD patients.

It has to be taken into account that for this study we did not have healthy controls available to compare our results to. It is therefore unclear whether GFD patients and RCD patients had increased IL-13 levels or ACD had reduced IL-13 levels. 

## 5. Conclusions

In conclusion our data show that IL-13 production is lower in the lamina propria of ACD patients, compared to GFD and in particular RCDII patients, suggesting that the immune responses in ACD and RCDII are differently regulated and that IL-13 may play a role as a proinflammatory cytokine in the pathogenesis of RCDII.

## Figures and Tables

**Table 1:** Patient characteristics and composition of leucocyte infiltrates.

	ACD	GFD	RCDII
	*N* = 4	*N* = 7	*N* = 9
Sex, % females	75.0%	71.4%	33.3%
Age, yrs	45.8 (22.2–75.3)	55.9 (35.3–72.0)	70.6 (41.7–76.2)
Villous atrophy, %	100%	0.0%	45.5%
Cell yield, 10^3^ IEL/biopsy	28.5 (7.5–58.0)	16.0 (2.1–113.0)	19.1 (5.8–62.4)
CD3^+^ IEL, % of CD45	99 (97–99)	96 (86–98)	21 (10–99)*
CD4^+^ IEL, % of CD45	5 (1–10)	3 (2–32)	5 (1–14)
CD8^+^ IEL, % of CD45	77 (59–86)	78 (65–90)	13 (5–69)*
CD16/56^+^ IEL, % of CD45	1 (0–3)	2 (1–11)	3 (0–24)
Aberr. IEL, % of CD45	0 (0-1)	2 (0–6)	66 (1–87)*
CD3^+^ LPL, % of CD45	40–43	21–60	25–38
CD4^+^ LPL, % of CD45	13–28	0–31	8–24
CD8^+^ LPL, % of CD45	10–16	2–29	4–14
CD16/56^+^ LPL, % of CD45	3-3	5–8	1–4
CD19^+^ LPL, % of CD45	4-5	1–8	3–10

For age and IEL data medians (5 percentile–95 percentile) are shown. For LPL data ranges are shown, since data for composition of LPL was available only in 2 ACD patients, 4 GFD patients, and 4 RCDII patients.

*Significantly lower percentage of CD3^+^ and CD8^+^ cells compared to ACD and GFD (due to high percentage of aberrant T-cells).

**Table 2:** Patient characteristics of RCDII patients.

	Sex	Age, yrs	Marsh	Treatment	Last treatment < 6 weeks before biopsy	Aberrant cells, % of CD45	Symbol Figures 1 and 2^d^
1^a^	M	68.1	IIIa	Chemotherapy, entocort	Yes	77%	*⚫*
2^c^	M	76.0	IIIa	2x cladribine	Yes	37%	■
3	F	72.8	IIIa	Cladribine	No	70%	▲
4	F	41.7	IIIb	None	No	87%	*▼*
5^b^	F	54.9	IIIc	6-TG	Yes	0.6%	*◆*
6	M	70.3	I	Cladribine, SCT	No	13%	○
7^c^	M	76.2	0	Cladribine	Yes	41%	□
8	M	72.9	I	SCT	No	73%	∆
9	M	70.6	I	Cladribine	No	66%	*◊*

^a^RCDII after successful treatment of enteropathy-associated T cell lymphoma.

^b^Enteropathy-associated T cell lymphoma was diagnosed when biopsy was taken.

^c^Patients 2 and 7 are the same patients before and after histological recovery.

6-TG: 6-thioguanine, SCT: stem cell transplantation.

^d^Corresponding symbol in Figures  1 and 2.

## Abbreviations

6-TG: 6-thioguanine
ACD: Active coeliac disease
RCD: Refractory coeliac disease
RCDII: Refractory coeliac disease type II
DDT: Dithio-DL-threitol
EDTA: Ethylen-diamin-tetra-acetic acid
GFD: Gluten-free diet
IL: Interleukin
IFN*γ*: Interferon gamma
LPS: Lipopolysaccharide
PMA: Phorbol myristate acetate
SCT: Stem cell transplantation
T_H_: T helper
TG2A: Transglutaminase-2 (= tissue transglutaminase) antibodies
TNF*α*: Tumor necrosis factor alpha.
